# Disseminated toxoplasmosis in an HIV positive patient in cART era

**DOI:** 10.1186/1471-2334-14-S4-P41

**Published:** 2014-05-29

**Authors:** Simona Claudia Cambrea, Sorina Dalia Carp, Stela Halichidis

**Affiliations:** 1 Ovidius University, Constanța, Romania; 2 Clinical Hospital of Infectious Diseases, Constanța, Romania

## 

Toxoplasmosis is a disease caused by the intracellular parasite *Toxoplasma gondii*. Toxoplasmosis is considered one of the most common cerebral opportunistic infections in HIV-AIDS patients. It develops when CD4 count falls below 100 cells/cmm, either from acute exposure to the parasite or from reactivation of latent infection. These patients may also develop extra-cerebral toxoplasmosis such as ocular toxoplasmosis and pulmonary diseases. Another clinical manifestation described in HIV-infected patients is disseminated toxoplasmosis which consists of fever, pulmonary infiltrates and sepsis-like syndrome.

We present a case of a 22 years old female patient diagnosed with HIV since her childhood. Over the years she was uncompliant to combined antiretroviral therapy (cART). After 16 years from her HIV diagnosis she presented an acute hepatitis type C with severe prolonged evolution, from which she slowly recover after a period of 3 months. After discharge she was well for about one month but came back with left hemiparesis difficulties in speech and visual disturbances. Also during this last hospitalization she presented a severe bronchopneumonia. After 20 days of hospitalization she died. Postmortem histopathological examinations revealed a disseminated toxoplasmosis involving multiple organ systems: central nervous system, lung, liver, spleen and lymph nodes. Lung examinations revealed bronchopneumonia due to multiple opportunistic coinfections with *T. gondii*, CMV and fungal infection. We noticed also an extensive ulcerated esophagitis of HSV and fungal etiology.

In immunosuppressed patients disseminated toxoplasmosis has a polymorphic clinical presentation requiring a more attentive investigation because it may hide also an involvement with other opportunistic infections. The syndrome of disseminated toxoplasmosis is affecting more than two organs and it is highly lethal in HIV positive patients.

